# The Bioenergetic Health Index: a new concept in mitochondrial translational research

**DOI:** 10.1042/CS20140101

**Published:** 2014-05-29

**Authors:** Balu K. Chacko, Philip A. Kramer, Saranya Ravi, Gloria A. Benavides, Tanecia Mitchell, Brian P. Dranka, David Ferrick, Ashwani K. Singal, Scott W. Ballinger, Shannon M. Bailey, Robert W. Hardy, Jianhua Zhang, Degui Zhi, Victor M. Darley-Usmar

**Affiliations:** *Mitochondrial Medicine Laboratory, University of Alabama at Birmingham, Birmingham, AL 35294, U.S.A.; †Department of Pathology, University of Alabama at Birmingham, Birmingham, AL 35294, U.S.A.; ‡Seahorse Bioscience, North Billerica, MA 01862, U.S.A.; §Division of Gastroenterology and Hepatology, Department of Internal Medicine, University of Alabama at Birmingham, Birmingham, AL 35294, U.S.A.; ∥Department of Veteran Affairs Medical Center, Birmingham, AL 35294, U.S.A.; ¶Department of Biostatistics, University of Alabama at Birmingham, Birmingham, AL 35294, U.S.A.

**Keywords:** aging, cardiovascular disease, haplotype, hepatotoxicity, neurodegenerative disease, oxidative stress, reserve capacity, BHI, Bioenergetic Health Index, ETC, electron transport chain, FCCP, carbonyl cyanide *p*-trifluoromethoxyphenylhydrazone, HNE, hydroxynonenal, LDA, linear discriminant analysis, mtDNA, mitochondrial DNA, OCR, oxygen consumption rate, RNS, reactive nitrogen species, ROS, reactive oxygen species

## Abstract

Bioenergetics has become central to our understanding of pathological mechanisms, the development of new therapeutic strategies and as a biomarker for disease progression in neurodegeneration, diabetes, cancer and cardiovascular disease. A key concept is that the mitochondrion can act as the ‘canary in the coal mine’ by serving as an early warning of bioenergetic crisis in patient populations. We propose that new clinical tests to monitor changes in bioenergetics in patient populations are needed to take advantage of the early and sensitive ability of bioenergetics to determine severity and progression in complex and multifactorial diseases. With the recent development of high-throughput assays to measure cellular energetic function in the small number of cells that can be isolated from human blood these clinical tests are now feasible. We have shown that the sequential addition of well-characterized inhibitors of oxidative phosphorylation allows a bioenergetic profile to be measured in cells isolated from normal or pathological samples. From these data we propose that a single value–the Bioenergetic Health Index (BHI)–can be calculated to represent the patient's composite mitochondrial profile for a selected cell type. In the present Hypothesis paper, we discuss how BHI could serve as a dynamic index of bioenergetic health and how it can be measured in platelets and leucocytes. We propose that, ultimately, BHI has the potential to be a new biomarker for assessing patient health with both prognostic and diagnostic value.

## INTRODUCTION

Complex and chronic diseases with underlying mechanisms involving dysfunctional metabolism are a growing healthcare problem in the developed world [1–3]. The availability of low-cost high-calorie foods in combination with a contemporary sedentary lifestyle presents a unique combination of risk factors with multiple evolving co-morbidities, which increasingly challenges our healthcare system especially in terms of prediction and management. Defining energetic health has become a necessity for healthcare in the 21st Century, and at the present time no clinical test is available to assess this parameter. We hypothesize that dysfunctional energetics associated with diabetes, cardiovascular disease, liver disease, cancer and environmental toxins can be dynamically assessed using a new parameter: the **B**ioenergetic **H**ealth **I**ndex (BHI) in patient populations. This approach has the potential to be used as the basis of personalized cell-based measurements to quantify bioenergetic health.

Our recent findings support an emerging concept that circulating leucocytes and platelets can serve as ‘the canary in the coal mine’ by acting as early sensors or predictive biomarkers of mitochondrial function under conditions of metabolic stress [4–8]. These studies prompted us to begin an integrated approach in cells isolated from human blood to establish a quantitative assay of mitochondrial function that will have the power to predict disease progression and response to treatment [9]. In the present Hypothesis paper, we introduce the BHI concept and its potential role in the emerging field of translational bioenergetics.

## EMERGING CONCEPTS IN BIOENERGETIC HEALTH

**Figure 1:**
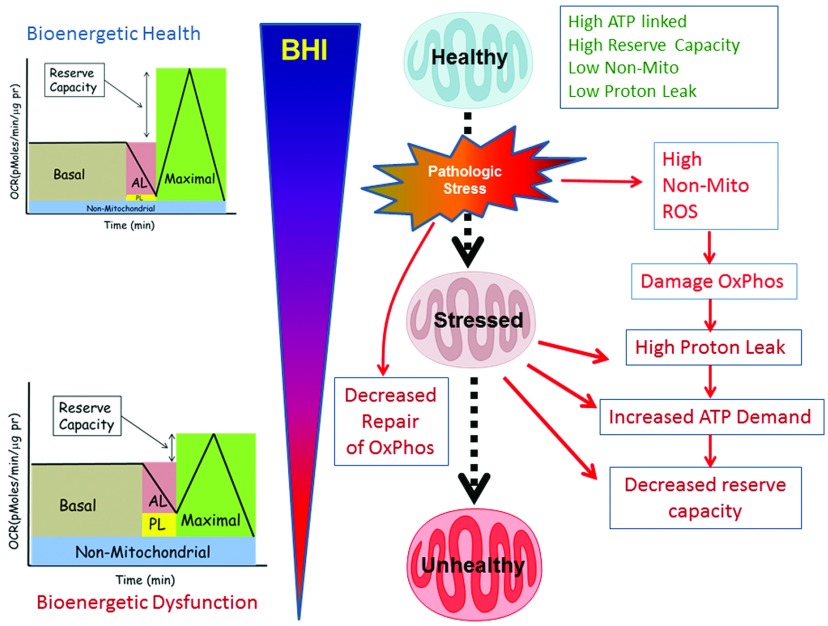
BHI as a dynamic measure of the response of the body to stress In this scheme, healthy subjects have a high BHI with a high bioenergetic reserve capacity, high ATP-linked respiration (AL) and low proton leak (PL). The population of mitochondria is maintained by regenerative biogenesis. During normal metabolism, a sub-healthy mitochondrial population, still capable of meeting the energetic demand of the cell, accumulates functional defects, which can be repaired or turned over by mitophagy. Chronic metabolic stress induces damage in the mitochondrial respiratory machinery by progressively decreasing mitochondrial function and this manifests as low ATP-linked respiration, low reserve capacity and high non-mitochondrial (e.g. ROS generation) respiration. These bioenergetically inefficient damaged mitochondria exhibit increased proton leak and require higher levels of ATP for maintaining organelle integrity, which increases the basal oxygen consumption. In addition, chronic metabolic stress also promotes mitochondrial superoxide generation leading to increased oxidative stress, which can amplify mitochondrial damage, the population of unhealthy mitochondria and basal cellular energy requirements. The persistence of unhealthy mitochondria damages the mtDNA, which impairs the integrity of the biogenesis programme, leading to a progressive deterioration in bioenergetic function, which we propose can be identified by changes in different parameters of the bioenergetics profile and decreasing BHI.

Mitochondria are highly sensitive to stress and respond dynamically to the changes in their cellular microenvironment. The macromolecules of the mitochondrion, including the respiratory chain complexes, are susceptible to oxidative damage which accompanies inflammation. We propose that failure to remove damaged mitochondria by mitophagy and replace them with healthy organelles can result in a progressive deterioration in bioenergetic function which precedes the onset of more severe clinical systems (Figure 1). The advent of high-throughput respirometry and the availability of specific mitochondrial inhibitors have stimulated the development of a method to obtain a bioenergetic profile for intact cells [10–12]. If bioenergetic health could be measured from these parameters at the ‘point of care’, it could have both diagnostic and prognostic value.

The initial reaction to the BHI concept might be ‘how can bioenergetics in circulating leucocytes and platelets act as a surrogate or marker of metabolic stress in specific tissues or organs?’ In part this question has been addressed because it is well established that diseases, including atherosclerosis, diabetes and neurodegeneration, are associated with deterioration in specific mitochondrial parameters and activities in cells throughout the body, including leucocytes and platelets [5,13–16]. Of particular interest is the fact that these pathologies are associated with increased oxidative stress and that mitochondria are both a source and target of ROS/RNS (reactive oxygen species/reactive nitrogen species). These insights, together with the advent of mitochondrial-targeted drugs, emphasize the need for quantitative methods to integrate these isolated measurements [17]. We propose that an individual's cellular bioenergetics can be measured in a clinical setting and used to generate a single integrated measure of bioenergetic health, i.e. the BHI.

## ESTABLISHING AND INTERPRETING THE CELLULAR BIOENERGETIC PROFILE

**Figure 2:**
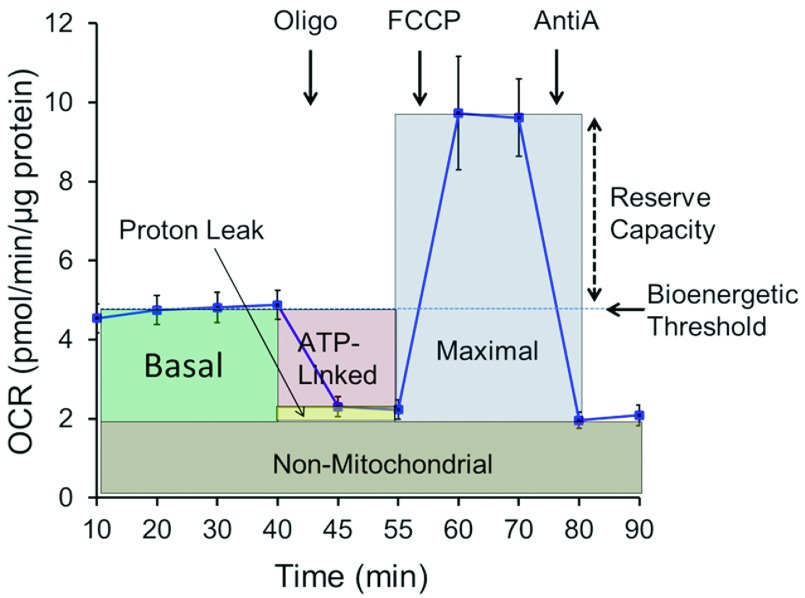
Cellular mitochondrial profile in human monocytes This assay defines cellular mitochondrial function using the well-defined inhibitors, oligomycin (Oligo), FCCP and antimycin A (AntiA) [12]. The interpretation of the different parameters defined by the assay is described in the accompanying text. Data is typically normalized to total protein or cell number in each well. Values are means±S.E.M., *n*=3–5.

Parameters from the cellular mitochondrial function assay (Figure 2) give insights into different aspects of mitochondrial function and below we discuss how these can be used to calculate the BHI. An important aspect of these mitochondrial parameters that can be measured from this assay is that they are potentially interactive and, taken together, can serve as a sensitive indicator of the response of cells to oxidative stress and the changing metabolic programmes associated with their role in inflammation.

### Basal oxygen consumption rate

The first measurement is the basal OCR (oxygen consumption rate) measured in the cells before injection of mitochondrial inhibitors. Changes in basal OCR in patients with disease relative to normal subjects can be interpreted with the information obtained from the rest of the profile.

### ATP-linked OCR and proton leak

After basal measurements are recorded, cells are exposed to oligomycin, which is an inhibitor of ATP synthase. By inhibiting proton flux through this enzyme, the increased proton gradient across the mitochondrial inner membrane prevents electron transport through Complexes I–IV. Oxygen consumption then decreases accordingly. The remaining rate of mitochondrial respiration represents proton leak, i.e. protons pumped during electron transport that result in oxygen consumption but not ATP production. An increase in the ATP-linked OCR would indicate an increase in ATP demand, whereas a decrease would indicate low ATP demand, a lack of substrate availability and/or severe damage to oxidative phosphorylation, which would impede the flow of electrons and result in a lower OCR.

An increase in apparent proton leak could be due to a number of factors including increased UCP (uncoupling protein) activity, damage to the inner mitochondrial membrane and/or ETC (electron transport chain) complexes. This results in the leakage of protons into the matrix and oxygen consumption in the absence of normal proton translocation across the inner mitochondrial membrane by Complexes I, III and IV, a process known as electron slippage. Increased calcium transport can also manifest as a change in proton leak. We have also shown that oxidative stress modifies the bioenergetic parameters and also increases ATP-linked oxygen consumption and proton leak [12].

### Maximal OCR and reserve capacity

An uncoupler, such as FCCP (carbonyl cyanide *p*-trifluoromethoxyphenylhydrazone), is next used to estimate maximal respiration; however, respiratory substrates are provided by cellular metabolism, which can be physiologically limiting [12]. A high FCCP-stimulated OCR compared with basal OCR indicates that the mitochondria are using less than the maximal rate of electron transport that can be supported by substrate supply from the cells. As shown in Figure 2, basal respiration can be considered a threshold below which the cell cannot sustain oxidative phosphorylation to meet energy demand. In support of this, we have demonstrated with mitochondrial inhibitors that reserve capacity is decreased by oxidative stress and, if this threshold activity cannot be met, glycolysis is then stimulated to meet the energetic needs of the cell [10,18–21]. The difference between the basal and maximal respiration is called the spare or reserve bioenergetic capacity [12,22]. The reserve capacity concept is well established in the literature. For example, it has been shown in the heart that, under an increased work load in the physiological range, mitochondria have a substantial ‘reserve capacity’, which is depleted under conditions of severe stress, including pressure overload or ischaemia [23,24]. More recently, we have shown that, under conditions of oxidative stress, the reserve capacity is depleted and if the threshold for the basal respiration is breached then cell death occurs [10,18,20,21,25–27].

Whether cells can utilize the maximal electron transport activity for ATP synthesis will depend on the capacity of the components of the oxidative phosphorylation system, including ATP synthase, which may be limiting. However, it is important to recognize that mitochondria in excitable cells, such as cardiomyocytes and neurons, are exposed to high fluxes of calcium and other ions, which will utilize the proton gradient and so increase the rate of oxygen consumption independent of ATP demand. Taken together, it is clear that reserve bioenergetic capacity is a cell- and context-dependent parameter intimately linked to bioenergetic health whether it is utilized for ATP synthesis or other mitochondrial functions. Importantly, a low maximal capacity could indicate decreased substrate availability or that mitochondrial mass or integrity is compromised. From a translational perspective, bioenergetic alterations in monocytes and lymphocytes are also linked to their changing biology during the progression of the inflammatory process [28,29].

### Non-mitochondrial OCR

This parameter is an index of oxygen-consuming processes that are not mitochondrial. In leucocytes, non-mitochondrial OCR is typically attributed to enzymes associated with inflammation, including cyclo-oxygenases, lipoxygenases and NADPH oxidases, and are regarded as negative indicators of bioenergetic health. We have shown that non-mitochondrial OCR varies and typically increases in the presence of stressors, including ROS and RNS, and it is well established that mitochondria are a target for the deleterious effects of these reactive intermediates [12,18].

## CALCULATION OF THE BHI

**Figure 3:**
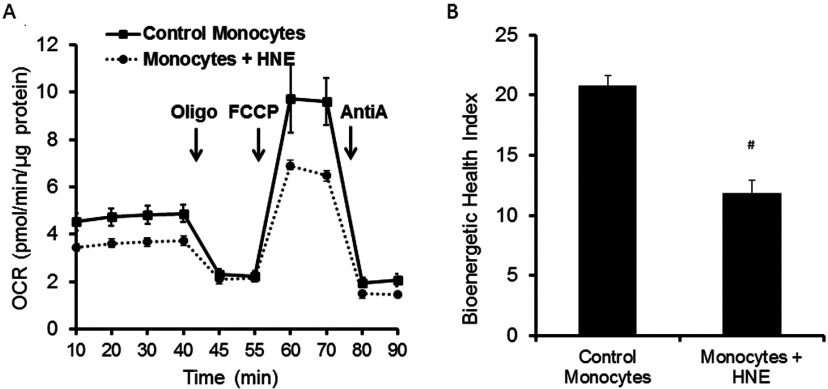
Change in the BHI of monocytes subjected to oxidative stress (**A**) The bioenergetic profiles of freshly isolated CD14^+^ monocytes from healthy volunteers were exposed to 4-HNE (20 μM for 1 h at 37°C) before the assay. AntiA, antimycin A; Oligo, oligomycin. (**B**) The BHI calculated using the mathematical relationship described in the text from the profile in (**A**) is demonstrated. Mean data (*n*=3–5 replicates) were plotted with ±S.E.M. (**A**) and +S.D. (**B**). #*P* ≤ 0.0001. All study protocols for collection and handling of human samples were reviewed and approved by the Institutional Review Board, University of Alabama at Birmingham.

In the present paper, we describe one of several possible variants for a BHI equation, which we designed using the standard statistical framework of LDA (linear discriminant analysis), which is consistent with the basic principles of bioenergetics. To test its responsiveness to oxidative stress, monocytes were exposed to the lipid peroxidation product 4-HNE (hydroxynonenal) as described below. We have described previously the effects of 4-HNE in cellular bioenergetics in a broad range of cell types [10,21,26,30]. In the 4-HNE example, a low BHI is associated with a lower reserve capacity, low ATP-linked respiration and increased proton leak (Figure 3). Eqn (1) shown below captures positive aspects of bioenergetic function (reserve capacity and ATP-linked respiration) and contrasts these with potentially deleterious aspects (non-mitochondrial oxygen consumption and proton leak). The first term in the numerator is the reserve capacity. The larger the value for reserve capacity the more effectively mitochondria can meet both the ATP needs of the cell and deal with increased energetic demand and ionic or metabolic stress [12].

(1) $$\mathrm{BHI}\;=\;\log\frac{{\left(\mathrm{reserve}\;\mathrm{capacity}\right)}^{a}\times {\left(\text{ATP-linked}\right)}^{b}}{{\left(\mathrm{non}\text{-}\mathrm{mitochondrial}\right)}^{c}\times {\left(\mathrm{proton}\;\mathrm{leak}\right)}^{d}}$$

The second term in the numerator, ATP-linked respiration, is a measure of the capacity of the cell to meet its energetic demands (Figure 1). For the denominator, the proton leak decreases mitochondrial efficiency with respect to ATP generation and is then a negative term. The final term in the denominator is the non-mitochondrial respiration. Non-mitochondrial oxygen-consuming processes are not well defined but in these cells they are predominantly those that originate from pro-oxidant and pro-inflammatory enzymes such as cyclo-oxygenases, cytochrome P450s or NADPH oxidases. As increased activity of these processes can damage mitochondria, we propose that the BHI will decrease under conditions of inflammation. The terms *a, b, c* and *d* are exponents (linear in log-space) which modify the relative weighting of the respiratory parameters.

To test the responsiveness of the BHI parameter to stress we exposed monocytes isolated from a healthy donor to the lipid peroxidation product 4-HNE. This reactive lipid intermediate has been found in a broad range of pathological conditions and damages mitochondria in cells by increasing proton leak and inhibiting electron transfer [17]. Shown in Figure 3(A) is the change in the mitochondrial profile following 4-HNE exposure and the corresponding change in BHI. In this example, the exponent parameters that modify reserve capacity, ATP-linked, non-mitochondrial and proton leak (*a*, *b*, *c* and *d*) were obtained by fitting the bioenergetic responses of monocytes to various concentrations of HNE using an LDA to determine the BHI function that maximizes the contrast between two conditions (Figure 3B). These data demonstrate that the BHI is responsive to oxidative stress in human monocytes. Weighting of these parameters can also be performed based on the relative biological significance or pathological relevance of individual parameters and differences in bioenergetic programmes between cell types. For example, if proton leak is revealed to contribute twice as much to cellular dysfunction as other parameters, disproportionate weighting would allow for a more specific and sensitive index.

In general, defects in the ETC will result in a lower BHI because of lower reserve capacity, ATP-linked respiration or increased uncoupling. It is important to note that cells which show a decrease in both reserve capacity and an increase in proton leak and non-mitochondrial respiration can still potentially provide sufficient ATP to meet the metabolic demands of the cell, but less efficiently. For this reason, the BHI has prognostic value because it can identify a progressive deterioration in bioenergetic health before the threshold at which failure to meet energy demand occurs.

## BHI IN LEUCOCYTES AND PLATELETS

Blood leucocytes and platelets are exposed to many soluble circulatory factors associated with metabolic stress and are, therefore, an ideal surrogate for determination of BHI in patients. Circulating cells, with the exception of erythrocytes and neutrophils, contain respiring mitochondria [9]. These cells sense and respond to systemic metabolic and inflammatory stressors and are therefore a functional biomarker in translational bioenergetics [5,14,31,32]. Importantly, circulating leucocytes and platelets have distinct life cycles, which have an impact on the cellular metabolic programmes they utilize for their evolving biological functions. Monocytes are phagocytic cells which survey the body for sites of inflammation and play an essential role in the innate immune system [33–35]. Bioenergetic changes in circulating monocytes could then reflect damage to mitochondria due to metabolic or oxidative stress, or the metabolic changes associated with inflammation.

Lymphocytes are a heterogeneous population of cells, which are normally in a quiescent state and are reliant on mitochondria to meet their energetic demands [36]. Activation of these cells is metabolically demanding because it must support clonal expansion, cytokine and antibody production, and is associated with an increase in both glycolytic activity and mitochondrial oxygen consumption [29,37–40]. Changes in bioenergetic function in patient populations can then reflect both metabolic stress and the changing role of these cells in immunity and inflammation.

Platelets are anuclear cytoplasmic fragments containing active mitochondria, which are released by resident megakaryocytes in the bone marrow. These cellular fragments have a short lifetime in the circulation (5–7 days) and, because their mitochondria cannot be replaced, they have frequently been used as a bioenergetic sensor in human subjects [14]. Under circulating conditions, both oxidative phosphorylation and glycolysis play a role in energy production in platelets but with minimal reserve bioenergetic capacity [41].

We have previously assessed the mitochondrial profile of these cell types and it is clear that each are unique in their mitochondrial and glycolytic programmes [9]. Consequently, interpretation of translational studies using isolated blood leucocytes and platelets should take into account that mitochondrial function differs between these cell types. The advantage of this approach is that platelets, lymphocytes and monocytes can act as differential sensors or biomarkers of mitochondrial dysfunction in different pathologies, thus increasing the breadth and diagnostic versatility of the BHI. For example, because the protein levels of Complexes III and IV are low in platelets, this cell type can serve as a sentinel for defects in these respiratory chain complexes compared with monocytes and lymphocytes, which have higher levels of these complexes [9]. It follows from these data that the BHI is likely to be different between leucocytes and platelets isolated from human blood.

## MITOCHONDRIAL VARIABILITY IN HUMAN SUBJECTS AND THE BHI

Mitochondrial dysfunction can promote altered energy expenditure and systemic inflammation that modifies susceptibility to energy-based pathologies associated with oxidative stress such as obesity and diabetes [42,43]. Mitochondrial proteins are encoded by both nuclear and mitochondrial genomes, and genetic changes in either the nucleus or mtDNA (mitochondrial DNA) can potentially alter mitochondrial bioenergetics and result in individual variation in the BHI within healthy populations. Genetic variations, either nuclear or mitochondrial, can also result in lower mitochondrial mass or function, which are exacerbated by aging, exposure to environmental toxins, lifestyle and disease risk factors [44–46]. Importantly, ‘normal’ genetic variation within mtDNA can be associated with changes in mitochondrial function and disease susceptibility that will be intertwined with cellular bioenergetics and inflammation [45,47–49]. Future studies investigating whether a relationship exists between the BHI and mtDNA haplotype or haplogroup are therefore of interest.

## DYNAMIC ASPECTS OF BHI MEASUREMENT

The role of metabolic stress in chronic disease development may be mediated through an inability to repair cellular damage from ROS (i.e. oxidative stress) that has been worsened by mitochondrial damage and heightened by systemic inflammation. In turn, this can damage bioenergetics in leucocytes and platelets, thereby allowing them to be sensors of bioenergetic health, as outlined in Figure 1 [28].

As discussed above, the critical factors which modify the BHI include changes in cellular metabolism that are responsive to changes in the environment (e.g. caloric intake and physical activity), those that can influence oxidant and/or inflammatory response, and racial differences in disease susceptibility due to differences in mitochondrial and nuclear genomes. This also suggests that the differential influence of factors such as genetic determinants, age, lifestyle and existing physiology/pathology in human health will be consolidated in the BHI for each individual. An important implication of this concept is that mitochondrial tests do not have to be localized to specific organs or tissues (e.g. liver or skeletal muscle), but can be assessed by an integrated test of bioenergetic function in cells isolated from an individual's blood. Refinements of the basic approach to measuring cellular energetics and parameters that could be included into the BHI calculation included glycolysis and the measurement of the response to different substrates.

## FUTURE OUTLOOK

In this Hypothesis paper our intent is to introduce the concept of the BHI and one possible equation for illustrative purposes. The benefit of using a data-driven definition of the BHI by fitting distinct bioenergetic parameters is that the general concept of the BHI can be adapted to different clinical settings. In this case we chose LDA for two reasons. First, it has a simple mathematical form [eqn (1)] and secondly, it can also be interpreted as conforming to Gaussian clustering. For the BHI defined over two conditions, e.g. normal compared with disease, each sample's BHI can be translated into the probability of the sample being normal, which also aids in the clinical application. As clinical data sets become available, other approaches to calculating the BHI can be explored. Indeed, the precise formulation of the BHI equation will require an extensive clinical trial with normal subjects and patients and the appropriate informatics analysis which we, and others, are in the process of obtaining.

The overall goal is to establish the BHI as a new universally deployed clinical test for assessing bioenergetic dysfunction especially early in disease progression before significant pathology and/or acutely prior to life-threatening conditions. If successful, the BHI test will then become an important approach to integrating personalized medicine with state-of-the-art translational bioenergetics.
